# The Influence of Competition Among *C. elegans* Small RNA Pathways on Development

**DOI:** 10.3390/genes3040671

**Published:** 2012-10-19

**Authors:** Jimmy J. Zhuang, Craig P. Hunter

**Affiliations:** Department of Molecular and Cellular Biology, Harvard University, Biological Laboratories, Room 3044, 16 Divinity Avenue, Cambridge, MA 02138, USA; E-Mail: jzhuang@mcb.harvard.edu

**Keywords:** microRNAs, endogenous siRNAs, enhanced RNAi, exogenous RNAi, RNA regulation

## Abstract

Small RNAs play a variety of regulatory roles, including highly conserved developmental functions. *Caenorhabditis elegans* not only possesses most known small RNA pathways, it is also an easy system to study their roles and interactions during development. It has been proposed that in *C. elegans*, some small RNA pathways compete for access to common limiting resources. The strongest evidence supporting this model is that disrupting the production or stability of endogenous short interfering RNAs (endo-siRNAs) enhances sensitivity to experimentally induced exogenous RNA interference (exo-RNAi). Here, we examine the relationship between the endo-siRNA and microRNA (miRNA) pathways, and find that, consistent with competition among these endogenous small RNA pathways, endo-siRNA pathway mutants may enhance miRNA efficacy. Furthermore, we show that exo-RNAi may also compete with both endo-siRNAs and miRNAs. Our data thus provide support that all known Dicer-dependent small RNA pathways may compete for limiting common resources. Finally, we observed that both endo-siRNA mutants and animals experiencing exo-RNAi have increased expression of miRNA-regulated stage-specific developmental genes. These observations suggest that perturbing the small RNA flux and/or the induction of exo-RNAi, even in wild-type animals, may impact development via effects on the endo-RNAi and microRNA pathways.

## 1. Introduction

Non-coding RNAs regulate a myriad of biological processes [1,2]. Two broad classes of highly conserved non-coding regulatory RNA pathways were discovered in the nematode *Caenorhabditis elegans*. First, microRNAs (miRNAs) were identified as transcribed RNAs corresponding to mutant loci that disrupted stage-specific developmental processes [3,4]. These small RNAs are complementary to nucleotide sequences in the 3’UTR of inferred target mRNAs and act to inhibit translation and/or mRNA target stability [3,4,5]. Subsequent genomic analysis identified scores of 22 nucleotide miRNAs, many broadly conserved in plants and animals [5,6]. Second, mechanistic investigation of the widely used technique of RNA interference (RNAi) revealed the existence of regulatory pathways that produce and use short interfering RNAs (siRNAs), whether derived from exogenous dsRNA (exo-siRNAs) or endogenous loci (endo-siRNAs) [7,8,9,10,11]. Investigations in other systems, including *Drosophila*, *Arabidopsis*, and mammalian cultures, have identified additional non-coding RNA pathways, including piwi-interacting RNA (piRNAs) and long non-coding RNA (lncRNAs), both of which have also been found in *C. elegans* [12,13,14]. The biological processes regulated by these small RNAs are quite extensive, including germline maintenance [13], chromosomal segregation [15], protection against transgenic parasites [16], multi-generational inheritance of RNAi signals [17], and developmental regulation [18].

Genetic screens have identified mutants in *C. elegans* with enhanced exo-RNAi responses (Eri mutants) [19,20,21,22,23]. Confoundingly, these mutants which enhance exo-RNAi disrupt endo-RNAi [21,22,23,24]. One proposed explanation is that endo-RNAi and exo-RNAi pathways compete for one or more limited common enzymatic resources, so that reduced endo-RNAi activity [11,25] results in greater flux of small RNAs through the exo-RNAi pathway [8,21]. Additional support for this competition model is provided by the identification of limiting RNAi components that are both necessary for the enhanced RNAi response and, when over-expressed, confer enhanced RNAi [21,26]. 

**Figure 1:**
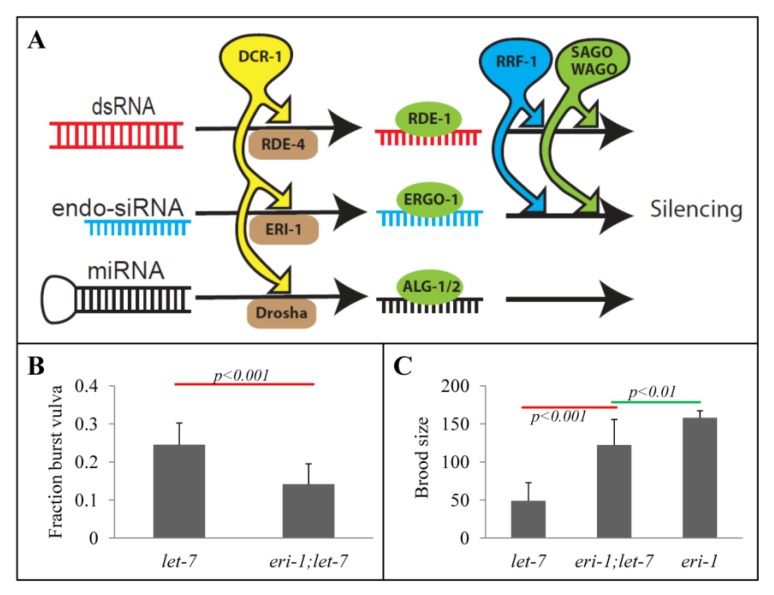
*eri-1* dependent *let-7* reduction-of-function phenotypes**. (A) **Schematic of proposed competing small RNA pathways. MicroRNAs, experimentally introduced double stranded RNAs (dsRNA), and endogenous short interfering RNAs (endo-siRNAs) were proposed [26] to compete for limiting shared resources, including the single *C. elegans* DICER homolog, *dcr-1*, the RNA-directed RNA polymerase RRF-1, and the secondary Argonautes (SAGOs), including the *C. elegans* specific worm Argonautes (WAGOs) that mediated siRNA-dependent silencing. The competition for limiting shared resources implies that reduced flux through one pathway allows for increased access to limiting resources for the other pathways. **(B)** Spontaneous vulva bursting rate of *let-7(n2853)* single (n = 9) and *eri-1(mg366);let-7(n2853)* double (n = 10) mutants at 15 °C. The average of n complete broods is shown. The *eri-1(mg366)* single mutant does not exhibit any burst vulva phenotype. **(C)** Average brood size of *let-7(n2853)* and *eri-1(mg366)* single mutants (n = 5) and *eri-1(mg366);let-7(n2853)* double mutants (n = 8) at 15 °C. Standard deviations are shown. *p*-values are calculated by *t*-test.

Although competition has only been inferred between endo- and exo-RNAi pathways, because *C. elegans* has only one *dicer* homolog—*dcr-1*—this competition model has been hypothesized to include other small RNA pathways, particularly the microRNA pathway [21,26]. This model proposes that various *dcr-1*-dependent non-coding RNAs compete for limited common enzymatic resources, including RNA-dependent RNA polymerases (RdRPs) that produce abundant secondary siRNAs and secondary siRNA binding Argonouates (SAGOs) [21] (Figure 1A). Consistent with this model, over-expression of some of these resources, specifically the secondary Agos SAGO-1 and SAGO-2, can enhance exo-RNAi [21,26]. However, direct evidence to support this broader competition model is lacking, especially with respect to small RNA pathways other than exo- and endo-RNAi. Moreover, many of these non-coding small RNAs, particularly miRNAs like *lin-4* and *let-7*, are developmentally regulated [3,4], which challenges simple interpretations of the extent of competition. For instance, the loss of one RNAi pathway may impact development, which in turn affects the production or stability of another pathway’s small RNAs. Such competition would not be for common RNAi resources, but rather reflect an indirect result of the physiological changes. The fact that weak alleles or maternal rescue of *dicer* causes sickly phenotypes in animals [23,27] may suggest that this is a realistic concern.

Here, we examine the relationship between endo-RNAi and miRNAi pathways and show that, although the *eri-1* mutant partially rescues the *let-7* mutant’s physiological defects, thus apparently enhancing residual *let-7* activity, Eri mutants also show increased expression of *let-7* targets *lin-14*, *lin-41*, and *daf-12*, thus appearing to simultaneously decrease *let-7* activity. Similarly, we show that engaging the exo-RNAi pathway, by exposing animals to *gfp* dsRNA, also appears to reduce the efficacy of both endoRNAi and miRNAi pathways. Finally, we observed that the relative expression of stage-specific developmental genes differs amongst small RNA pathway mutants, suggesting that in addition to competition for common limiting RNAi resources, perturbing small RNA flux also impacts developmental regulation. This may have large and indirect effects on the activity of endogenous small RNA pathways.

## 2. Results and Discussion

### 2.1. Rescue of let-7 Phenotypes by the eri-1 Mutant

To determine whether the *eri* pathway competes with the miRNA pathway (Figure 1A), we sought to determine whether, like the genetic interaction between the exo-RNAi and endo-RNAi pathways, similarly disrupting the endo-RNAi pathway could abrogate miRNA developmental defects. To detect changes in miRNA pathway efficacy, we scored fertility and vulva integrity [28] of *let-7(n2853)* single and *let-7(n2853);eri-1(mg366)* double mutant animals. The *n2853* allele is a partial loss-function mutation that remains viable at 15 °C [4]. We found that the *eri-1;let-7* double mutant exhibited a significant reduction in the burst vulva phenotype (Figure 1B), and an increase in brood size compared to the *let-7(n2853)* single mutant (Figure 1C). Although the rescue was incomplete, these results indicate a genetic interaction between these two small RNA pathways. Because the disruption of endogenous siRNAs seems to suppress the *let-7* phenotypes, the implication is that the endo-siRNA pathway may compete with the miRNA pathway. To our knowledge, this is the first reported genetic interaction between these two pathways.

### 2.2. eri-1 Mutant Worms Have Higher Expression of let-7 Regulated Genes at Mid-L4

**Figure 2:**
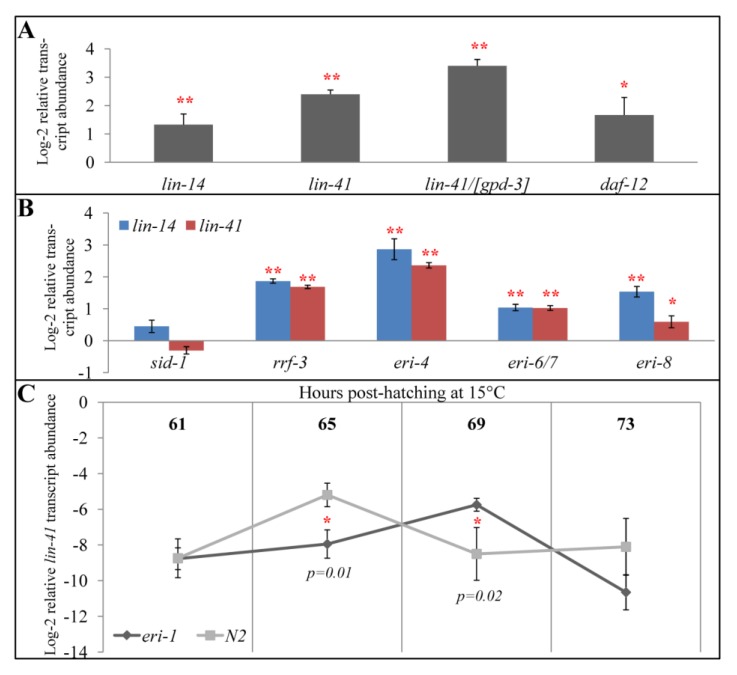
Expression of *let-7*-regulated transcripts in endo-RNAi mutant backgrounds. **(A)*** bli-1* normalized abundance of the *let-7*-repressed *lin-14*, *lin-41*, and *daf-12* transcripts in *eri-1(mg366)* mid-L4 animals (69 hours post hatching at 15 °C) relative to N2 wild type. *gpd-3* normalized *lin-41* transcripts in *eri-1(mg366)* mid-L4 animals relative to N2 is indicated by “*lin-41*/[*gpd-3*]”. **(B)*** bli-1* normalized abundance of the *lin-14* and *lin-41* transcripts in the indicated endo-RNAi and control (N2 and *sid-1(qt9)*) mid-L4 animals. **(C) **Developmental analysis of *lin-41* transcripts during the L4-stage in *eri-1(mg366)* and N2 strains, normalized to *gpd-3*. Although *lin-41* transcripts become rare, Ct values remained reliable through 69 hours post hatching (≤30); but by 73 hours post hatching, Ct values were greater than 34 and therefore considered undetectable and unreliable [37,38]. Error bars indicate standard deviation in **(A) **and** (B)**, and 95% confidence interval in **(C)**. * indicates *p < 0.05*; ** indicates *p < 0.01*; no asterisks indicate statistical insignificance. *p*-values are calculated by *t*-test.

To determine whether the observed genetic interaction reflects enhanced miRNA efficacy (rather than indirect effects, for example loss of a specific siRNA), we measured the abundance of *let-7* regulatory targets, which increase in abundance in *let-7* mutants [4]. If *eri-1*-mediated repression of *let-7* is via reduced competition and consequently the production of higher *let-7* levels or more effective use of *let-7* miRNA, then the target genes should decrease in abundance in the *eri-1* mutant relative to wild type. However, if the phenotypic suppression of *let-7(2853)* in an *eri-1* mutant background is indirect, then no effect on *let-7* target mRNA levels is expected. An advantage of assaying target mRNA levels is that the competition model does not make any predictions about which stage of small RNA processing or activity the competition occurs. This means the production, stability, or even efficacy of small RNAs could be rate-limiting. A complication is that *let-7* regulates temporally expressed genes, thus it is critical to measure RNA levels from precisely staged animals. Therefore, RNA was extracted from mid-fourth larval stage (L4) worms hatch-synchronized to within 1 hour and then raised at 15 °C for an additional 69 hours. As an additional level of control, we normalized gene expression to *bli-1*, an L4-specific collagen gene that is not known to be regulated by endogenous small RNAs [29]. Thus, in essence, developmental time is redundantly staged, by both “human time”—69 hours post-hatching—as well as “worm time”—*bli-1* mRNA expression. Surprisingly, we observed that the *let-7*-regulated genes *lin-14*, *lin-41*, and *daf-12* [5] all showed increased expression in an *eri-1* mutant background (Figure 2A). To ensure that this is not unique to *bli-1* normalization, we assayed for expression levels of *lin-41* normalized to the glyceraldehyde 3-phosphate dehydrogenase *gpd-3*, a stably expressed housekeeping gene that is commonly used for *C. elegans* mRNA normalization [30]. Again, *lin-41* showed increased expression in an *eri-1* mutant background (Figure 2A), suggesting that this observation is not a normalization artifact. Thus, *let-7*-regulated genes are affected by endo-siRNA depletion, but rather than an increase in *let-7* activity, we observed an increase in target mRNA levels, which suggests an apparent decrease in *let-7* efficacy.

### 2.3. All Eri Mutants Have Higher Expression of let-7-regulated Genes at Mid-L4

Biochemically, the endo-siRNA pathway is diverse and includes RdRPs (RRF-3), siRNases (ERI-1), RNA binding Tudor domain proteins (ERI-5), helicases (ERI-4, ERI-6/7), and Agos (ERI-8). To determine whether the observed increase in *let-7* target mRNA levels is specific to *eri-1(mg366)*, or represents a more general effect of reduced function in the endo-siRNA pathway, we determined the expression levels of *lin-14* and *lin-41* in *rrf-3(pk1426)*, *dcr-1/eri-4(mg375)*, *eri-6/7(tm1917)*, and *ergo-1/eri-8(gg100)* mutant backgrounds. Consistent with our *eri-1* observations, these mutants also showed increased expression of the two *let-7* targets (Figure 2B), suggesting that a functional endo-siRNA pathway is important for *let-7* efficacy. 

Included in these experiments were three independent controls. First, as expected, *lin-14* and *lin-41* expression increased between 20 to 60 fold in *let-7(n2853)* mutants compared to N2 wild type. Similarly, the viable *dcr-1(mg375)* allele showed a very strong upward effect on the *let-7* target mRNA levels, as would be expected for a mutation that directly affects miRNA biogenesis [23]. Finally, as a negative control, we assayed *lin-14* and *lin-41* expression levels in a *sid-1(qt9)* background. Because *C. elegans* miRNAs seem to act cell autonomously [31], their activity should not require the systemic double-stranded RNA channel SID-1. As expected, *lin-14* and *lin-41* expression levels were indistinguishable between *sid-1(qt9)* and N2 animals (Figure 2B). 

### 2.4. eri-1 Mutants May Have Delayed Down-regulation of lin-41

Our findings thus far indicate that mutations in the endo-siRNA pathway phenotypically suppress *let-7*, consistent with the proposed competition model, whereas the effect on the *let-7* target mRNAs was opposite to expectations, showing an increase instead of a decrease (Figure 2A, B). While there are many possible explanations, including post-transcriptional silencing versus translational repression, one possibility that should be considered and that can be directly tested is that developmental time is affected in the endo-RNAi mutants. *let-7* acts as a temporal developmental switch, down-regulating target genes to control stage-specific differentiation. Therefore, minor effects on *let-7* temporal activity would be amplified. Although we attempted to control for this possibility by normalizing mRNA levels to the L4-specific collagen *bli-1*, the apparent increase in the abundance of *let-7* target transcripts in endo-RNAi mutants may reflect a developmental time point before *let-7* accumulates to effective levels. That is, the endo-RNAi mutants may slow worm developmental time relative to human time, delaying both accumulation of *let-7* and the predicted transition to L4 gene expression patterns. To test this hypothesis, we collected RNA from hatch-synchronized worms raised at 15 °C for 61, 63, 69 and 73 hours to measure *lin-41* levels prior, during, and after *let-7* regulation. 

Consistent with previous observations, in a wild type background, *lin-41* levels were high at the beginning of the L4 stage (hours 61 to 65), but dropped to undetectable levels at later L4 stages (hours 69 to 73) [4] (Figure 2C). Interestingly, in an *eri-1(-)* background, *lin-41* transcript levels remained at the higher level for at least an additional four hours. In wild type, the decrease in *lin-41* levels was detectable at 65 hours, whereas in *eri-1* mutants, the decrease was first detectable at 69 hours. This seems to suggest that there was a temporal delay in the decrease of *lin-41* expression (Figure 2C). Although the number of time points is limited, these observations are consistent with a hypothesis that developmental time runs slower in endo-RNAi mutants. Consequently, miRNAs would not be as effective at a particular human time point (*i.e.*, hour 69) because the *eri-1(-)* worms haven’t reached the equivalent worm time (mid-L4) for a maximum *let-7* efficacy. 

However, other trivial explanations may also fit the data, including a very likely possibility that data points at hours 65 and 69 were noise fluctuations in two genes’ expression between the two strains (Figure 2C). Unfortunately, we were unable to distinctly quantify differences in *let-7* transcripts itself because of its relative rarity and the relatively subtle differences in expression between mutants, which prevented us from drawing additional support for this delayed development hypothesis.

### 2.5. Perturbations to Small RNA Pathways Affect Developmental Timing

**Figure 3:**
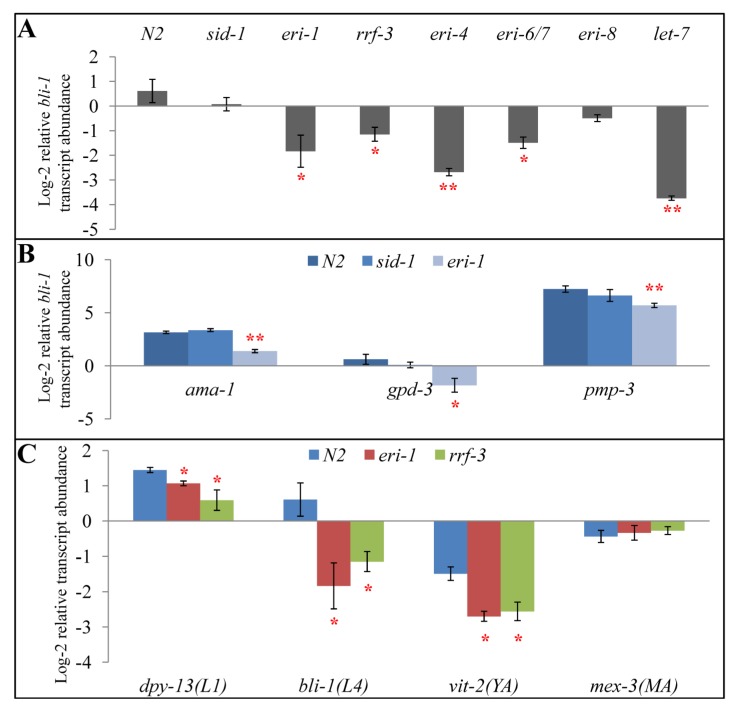
Abundance of developmentally regulated transcripts in endo-RNAi mutants. **(A) ***gpd-3* normalized *bli-1* transcript abundance at mid-L4 (69 hours post hatching at 15 °C) in the indicated mutants. **(B)** Relative *bli-1* transcript levels in *eri-1(mg366)* compared to N2 and *sid-1(qt9)* at mid-L4, normalized to the three indicated housekeeping genes. **(C) **Relative expression of the indicated developmental stage-specific genes at the denoted stages in *eri-1(mg366)* and *rrf-3(pk1426)* mutants compared to N2. At 15 °C, L1 worms had their RNA extracted 22 hours post-hatching, L4 worms had their RNA extracted 69 hours post-hatching, young adult (“YA”) worms had their RNA extracted 80 hours post-hatching, and mature adults (“MA”) worms had their RNA extracted 100 hours post-hatching. Error bars indicate standard deviation. * indicates *p < 0.05*; ** indicates *p < 0.01*; no asterisks indicate statistical insignificance. *p*-values are calculated by *t*-test.

Therefore, to test, confirm, and generally expand the hypothesis that development is slowed in endo-RNAi mutants, we performed real-time PCR to measure the relative expression levels of a variety of stage-specific mRNAs relative to stable housekeeping genes. The collagen *bli-1* is transcribed specifically during the L4 stage [29]. Consistent with our hypothesis, *bli-1* transcript levels relative to *gpd-3* transcript levels—which are transcribed stably throughout development [30,32]—were reduced in all the endo-RNAi mutants compared to N2 wild type (Figure 3A). As with *let-7* target genes, the systemic RNAi mutant *sid-1(qt9)* had no detectable effect. Furthermore, both *let-7(n2853)* and *dcr-1/eri-4(mg375)* mutants, which are known to have significant sickness [4,5,23,33], showed much larger *bli-1* versus *gpd-3* differences than the other endo-RNAi mutants (Figure 3A). Finally, the *ergo-1/eri-8(gg100)* allele, which causes the least sterility and brood size reduction [33] of the tested endo-RNAi mutants, showed only a small and not statistically significant reduction in relative *bli-1* transcript levels (Figure 3A). 

These findings suggests that either endo-RNAi mutants slow development leading to lower *bli-1* levels 69 hours post hatching, or that these mutants affect metabolism leading to an increase in *gpd-3* levels. To control for this second possibility, we used two additional stably expressed housekeeping genes: *ama-1* (a measure of RNAPII levels) and *pmp-3* (a measure of peroxisome activity) [32], in addition to *gpd-3*, to normalize *bli-1* transcript levels (Figure 3B). We found that *eri-1* mutants have lower expression of *bli-1* relative to all three stable housekeeping markers compared to N2 and *sid-1(qt9)* mutants. This analysis confirms that at 69 hours post hatching, *eri-1(mg375)* worms compared to N2 and *sid-1(qt9)* worms likely exhibit slowed development. Therefore, these results further suggest that the endo-RNAi mutants reduce the accumulation of L4 specific *bli-1* transcript due to a delay in development. 

Combined with the measurements of *lin-41* transcripts (Figure 2C), these results seem to suggest that endo-RNAi mutants delay development to the mid-L4 stage. To determine whether other developmental transitions are delayed in endo-RNAi mutants, we measured in both *eri-1* and *rrf-3* mutants the relative transcript levels of the L1 specific collagen *dpy-13* [34] 22 hours after hatching, the yolk protein gene *vit-2* [35] in young adult 80 hours after hatching, and the germline specific RNA binding protein *mex-3* [36] in mature adults 100 hours after hatching. We found that when compared to N2, the *eri-1* and *rrf-3* mutants showed significantly lower *dpy-13*, *bli-1*, and *vit-2* relative expression levels (Figure 3C). However, the expression levels of the germline specific *mex-3* were not affected in either mutant (Figure 3C). These results confirm that endo-RNAi mutants display mild developmental delays throughout development, but that by the mature adult stage 24 hours past the last molt, the effect is either no longer distinguishable or that *mex-3* is not a sufficiently sensitive temporal marker.

### 2.6. Impact of Developmental Timing Disturbances on Expression Studies

The developmental delay associated with endo-RNAi mutants may introduce a confounding factor for gene expression and RNAi phenotypic studies. Specifically, we detected two to four fold differences in measured developmental transcript levels in endo-RNAi mutants relative to wild type (Figure 3A). Because these differences are likely associated with developmental delays (Figure 2C), small changes in gene expression may not reflect a causal relationship between small RNA regulation and transcript abundance. Thus, careful calibration for developmental markers may be required before assigning significance to gene expression changes less than 4-fold among the many current *C. elegans* gene-expression datasets performed in endo-RNAi mutants [11]. In addition, many exoRNAi studies use endo-RNAi mutant backgrounds for their enhanced exo-RNAi phenotype, possibly further subtly confounding the findings.

### 2.7. Exogenous RNAi Causes Higher Expression of Some let-7- and rrf-3- Regulated Genes

**Figure 4:**
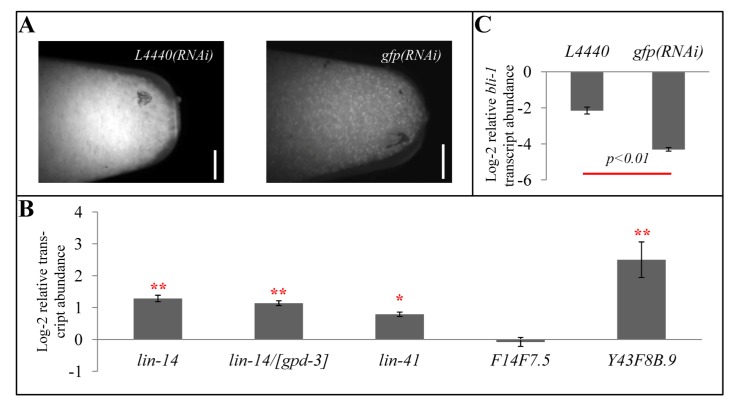
The effect of exogenous RNAi on transcript abundance of miRNA- and endo-RNAi-regulated genes. **(A)** Photomicrograph of ~200 µL of *sur-5::gfp* animals grown on control (empty L4440 vector) or *gfp(RNAi)* bacteria prior to RNA extraction. Scale bar indicates 1 mm. **(B) ***bli-1* normalized relative *lin-14*, *lin-41*, *F14F7.5*, and *Y43F8B.9* transcript levels in mid-L4 (69 hours post hatching at 15 °C) *gfp(RNAi)*-treated *sur-5::gfp* animals compared to L4440 vector-fed *sur-5::gfp* animals. *gpd-3* normalized *lin-14* transcripts in mid-L4 *gfp(RNAi)*-treated *sur-5::gfp* animals compared to L4440 vector-fed *sur-5::gfp* animals is indicated by “*lin-14*/[*gpd-3*]”. **(C)*** gpd-3* normalized relative *bli-1* transcript levels in mid-L4 *gfp(RNAi)*-treated *sur-5::gfp* animals compared to L4440 vector-fed *sur-5::gfp* animals. * indicates p < 0.05; ** indicates p < 0.01; no asterisks indicate statistical insignificance. *p*-values are calculated by *t*-test.

The competition model for interactions among the small RNA pathways further predicts that engaging the exo-RNAi pathway will reduce available resources for microRNA-regulated gene expression, resulting in increased target gene expression. To test this prediction, we compared the expression of *lin-14* and *lin-41* between control animals and the same strain exposed to dsRNA. Specifically, a strain expressing the ubiquitous *sur-5:gfp* transgene was grown on either empty vector L4440 bacteria (control) or *gfp*-dsRNA expressing bacteria (exo-RNAi(+)) (Figure 4A). RNA was then extracted from mid-L4 (69 hour hatch-synchronized) worms raised at 15 °C on each of the bacteria. Quantitative PCR analysis showed that the *lin-14* and *lin-41* transcripts, normalized to *bli-1*, were both elevated when this strain was grown on *gfp* RNAi food (Figure 4B). Similar results are obtained when transcripts levels were normalized to *gpd-3* (Figure 4B).

To similarly test for competition between the exo-RNAi pathway and the endo-RNAi pathway, we measured the transcript levels of the recently identified *rrf-3*-siRNA targets *F14F7.5* and *Y43F8B.9* [11]. As a control, we found these transcripts to be nearly sixty-fold and nearly eight-fold, respectively, more abundant in *rrf-3(pk1426)* mutant than in wild type mid-L4 worms, similar to previously published findings [11]. Consistent with the competition model, *gfp(RNAi)*-treated animals exhibited higher expression levels of the *Y43F8B.9* gene (Figure 4B). However, we found that *gfp(RNAi)*-treated animals did not exhibit significant differences in expression levels of *F14F7.5* compared to vector L4440-treated animals (Figure 4B).

Our results support expanding the competition model to include the microRNA pathway. We hypothesize that mounting an exo-RNAi response reallocates common resources necessary for miRNA and endo-RNAi pathways towards the exo-RNAi pathway, resulting in less effective repression of *let-7* and *rrf-3* target genes. To our knowledge, this is the first reported investigation of the interaction between exoRNAi and miRNA functions. Furthermore, while prior studies of the competition between endoRNAi and exoRNAi focused on the enhanced exoRNAi phenotype of endo-RNAi mutants, we presented data showing that engaging the exo-RNAi pathway compromises the silencing of at least one endoRNAi target. 

Interestingly, engaging the exo-RNAi pathway also reduced a worm’s relative *bli-1* expression at mid-L4 (Figure 4C). This result is consistent with delayed development associated with reducedendo-RNAi activity (Figure 2C). We therefore propose that reduced small RNA flux through endo-RNAi pathways enhances miRNA and exo-RNAi pathways by enabling greater access to limiting resources as well as independently delaying development. 

### 2.8. Chicken and Egg: Development and Small RNAs?

Our findings show that perturbed small RNA flux delays development. This may be caused by interference with miRNA-mediated regulation of the heterochronic genes that control temporal transitions, or the delayed expression of the heterochronic genes may simply reflect non-specific developmental delays. In either case the effects are likely to recursive, such that small initial differences are amplified as development progresses. As more small RNA deep sequencing data and analysis becomes available, it will be intriguing to analyze development from the perspective of small RNAs as master regulators. From an experimental standpoint, it is already important to consider such assumptions. Our data strongly suggest that endo-RNAi pathways are important for development, and although subtle, the effects may confound gene expression studies.

## 3. Materials and Methods

### 3.1. Strains

The following strains were used: (FX1917) *eri-6/7(tm1917)*, (GR1373) *eri-1(mg366)*, (HC195) *nrIs20 (sur-5::NLS-GFP)*, (HC196) *sid-1(qt9)*, (HC793) *eri-1(mg366);let-7(n2853)*, (MT7626) *let-7(n2853)*, (NL2099) *rrf-3(pk1426)*, (YY168) *ergo-1/eri-8(gg100)*, and (YY470) *dcr-1/eri-4(mg375)*. All strains and assays were maintained and performed at 15° C as previously described [39].

### 3.2. gfp(RNAi)

*gfp(RNAi)* assays were performed as previously described [40]. Bacteria engineered to express dsRNA against *gfp* were prepared as previously described [41].

### 3.3. Reverse Transcription Real-time PCR

Hatch**-**synchronized to within 1 hour, worms from NG large plates, *gfp(RNAi)* plates, or L4440 vector plates grown at 15 °C for needed number of hours were pooled, washed extensively (M9) and then allowed to swim for 20 minutes to clear gut content. RNA was isolated with proteinase K (Omega) followed by phenol:chloroform extraction (Amresco). The RNA pellets were subjected to DNase I (Roche) treatment, and cleaned by RNeasy (Qiagen) per manufacturer’s instructions. All initial RNA input concentrations were normalized to ~30−200 ng/µL.

Reverse transcription was performed using 20 µL reactions of ~150–600 ng of input RNA by Thermoscript RT (Invitrogen), using gene specific RT primers (available upon request). cDNA quantification was performed using 2 µL of the RT reaction in a 50 µL QuantiTect SYBR Green (Qiagen) reaction with nested PCR primers. The PCR reaction cycles were: 15 minutes 95 degrees, 15 seconds 94 degrees, 30 seconds 52 degrees, 1 minute 72 degrees, read, cycle to step 2 for 45 cycles, using an Eppendorf Mastercycler Realplex4 and Noiseband quantification. Subsequent analysis was performed using the ΔCT approach for expression normalized to another gene, or the ΔΔCT approach for expression normalized to another gene and then relative to wild type’s expression [42]. 

Three to five biological replicates of worms were combined for an RNA prep. This RNA was then quantified in triplicates (error bar generation), and repeated two to four times (representative shown), to determine the precision of relative RNA abundance, similar to prior small RNA qPCR experiments [25].

## 4. Conclusions and Future Scope

Recent publications report on competitive regulation schemes akin to the *C. elegans* small RNA competition model [8] for RNAs in mammalian systems [43,44,45,46,47]. These reports identify competitive endogenous RNAs (ceRNAs) that soak up miRNAs involved in tumorigenicity [43]. The use of miRNA profiling to diagnose cancer is becoming common, thus our findings in *C. elegans*, which suggest that developmental timing is intricately tied to small RNA expression, provides a cautionary caveat to these approaches. That is, while the functions of these ceRNAs are robustly demonstrated *in vitro*, when and how they are expressed *in vivo* for their putative regulation of miRNAs is not fully known. It is reasonable to assume that these ceRNAs are spatially and temporally regulated. Defects in tumors growth can impact small RNA expression, thus attributing causal relationships when developmental factors may indirectly affect both miRNAs and ceRNAs should be done with caution.

Therapeutic delivery of small RNAs has been a commercial goal of RNAi for many years [48]. Because the mammalian RNAi response apparently lacks an amplified secondary response [49], and the *C. elegans* limiting RNAi resources are largely involved in the biogenesis or execution of the secondary response, analogies to the *C. elegans* competition model were deemed inapplicable. However, our observation that engaging the exoRNAi pathway via experimental dsRNA can cause developmental delays suggests that the indirect aspect of the *C. elegans* small RNA competition can still serve as a model. Off-target effects have long been assumed to result from promiscuous siRNA binding, but our results suggest that perturbed small-RNA flux may also indirectly misregulate biological, processes, including development. 
